# Mutagenic analysis of actin reveals the mechanism of His161 flipping that triggers ATP hydrolysis

**DOI:** 10.3389/fcell.2023.1105460

**Published:** 2023-03-16

**Authors:** Mitsusada Iwasa, Shuichi Takeda, Akihiro Narita, Yuichiro Maéda, Toshiro Oda

**Affiliations:** ^1^ Graduate School of Informatics, Nagoya University, Nagoya, Japan; ^2^ Structural Biology Research Center, Graduate School of Science, Nagoya University, Nagoya, Japan; ^3^ Research Institute for Interdisciplinary Science (RIIS), Okayama University, Okayama, Japan; ^4^ Graduate School of Science, Nagoya University, Nagoya, Japan; ^5^ Faculty of Health and Welfare, Tokai Gakuin University, Kakamigahara, Japan

**Keywords:** MD simulation, actin, water dynamics, ATP hydrolysis, X-ray structure, baculovirus expression

## Abstract

The dynamic assembly of actin is controlled by the hydrolysis of ATP, bound to the center of the molecule. Upon polymerization, actin undergoes a conformational change from the monomeric G-form to the fibrous F-form, which is associated with the flipping of the side chain of His161 toward ATP. His161 flipping from the gauche-minus to gauche-plus conformation leads to a rearrangement of the active site water molecules, including ATP attacking water (W1), into an orientation capable of hydrolysis. We previously showed that by using a human cardiac muscle α-actin expression system, mutations in the Pro-rich loop residues (A108G and P109A) and in a residue that was hydrogen-bonded to W1 (Q137A) affect the rate of polymerization and ATP hydrolysis. Here, we report the crystal structures of the three mutant actins bound to AMPPNP or ADP-P_i_ determined at a resolution of 1.35–1.55^ ^Å, which are stabilized in the F-form conformation with the aid of the fragmin F1 domain. In A108G, His161 remained non-flipped despite the global actin conformation adopting the F-form, demonstrating that the side chain of His161 is flipped to avoid a steric clash with the methyl group of A108. Because of the non-flipped His161, W1 was located away from ATP, similar to G-actin, which was accompanied by incomplete hydrolysis. In P109A, the absence of the bulky proline ring allowed His161 to be positioned near the Pro-rich loop, with a minor influence on ATPase activity. In Q137A, two water molecules replaced the side-chain oxygen and nitrogen of Gln137 almost exactly at their positions; consequently, the active site structure, including the W1 position, is essentially conserved. This seemingly contradictory observation to the reported low ATPase activity of the Q137A filament could be attributed to a high fluctuation of the active site water. Together, our results suggest that the elaborate structural design of the active site residues ensures the precise control of the ATPase activity of actin.

## 1 Introduction

Actin is one of the most abundant proteins in eukaryotic cells and plays essential roles in various cellular activities, including locomotion, division, and transcription control (Le Clainche and Carlier, 2008; Pollard and Cooper, 2009; Kelpsch and Tootle, 2018). Actin has two assembly states: globular monomeric actin (G-actin) and fibrous polymerized actin (F-actin). F-actin, a linear assembly of G-actin, can be arranged in parallel, anti-parallel, and mesh-like networks. F-actin serves as a building block for cellular superstructures *in vivo*, including stress fibers, stereocilia rootlets, and contractile rings. These actin-based arrays are formed and maintained by several actin-binding proteins.

The transition between G- and F-actin is coupled with the actin ATPase reaction (Fujiwara et al., 2018; Lappalainen et al., 2022). Under physiological conditions, G-actin, which binds with ATP, polymerizes spontaneously into filaments. Polymerization induces the hydrolysis of actin-bound ATP to ADP-P_i_, followed by the slow release of γ-phosphate, which enhances the depolymerization of the filament back to G-actin. In addition, the ADP-bound F-actin molecule has a high affinity for actin-depolymerizing factors such as cofilin, resulting in accelerated depolymerization (Maciver et al., 1991; Carlier et al., 1997). ATPase activity is key to controlling the turnover cycle between G- and F-actin and the dynamic turnover of cellular arrays. Therefore, it is important to understand the coupling through the conformational transition from G- to F-actin.

The atomic structure is essential to understand the mechanism of actin ATPase in F-actin. The first crystal structure of actin was solved in 1990 as a complex with DNase I (Kabsch et al., 1990) which consists of two large domains, twisted ∼20° relative to each other, with the active site for ATP hydrolysis located in the cleft between the two domains. The G-actin structure led to a model of the arrangement and orientation of actin molecules within filaments (Holmes et al., 1990). In 2009, we found that the G- to F-actin transition causes relative rotation of the two large domains, thereby flattening the actin molecule (Oda et al., 2009). The twisted G-actin conformation and flattened F-actin conformation are called the G-form and F-form, respectively (Oda et al., 2019). Cryo-EM F-actin models at ∼3 Å resolution have revealed detailed intra- and inter-subunit structures within the filament (Merino et al., 2018; Chou and Pollard, 2019). The G- to F-form transition scarcely affected the structure of the active site except for two residues, Gln137 and His161. Notably, His161 rotates (flips) its side chain toward ATP, suggesting its involvement in hydrolysis. Gln137 and His161 have been shown to be involved in the hydrolysis of actin from G-actin crystal structures (Vorobiev et al., 2003).

Recently, we found that in crystals, the first domain of fragmin (F1) belonging to the gelsolin superfamily could fix the actin conformation in the F-form without polymerization (Kanematsu et al., 2022). Using this technique, we solved the structures of F-form actin bound to AMPPNP (ATP analog), ADP-P_i_, and ADP at high resolution (∼1.15 Å) and revealed the mechanism of actin ATP hydrolysis using QM/MM calculations (Kanematsu et al., 2022). Generally, the distance between the γ-phosphorous atom of ATP (P^G^) and a water molecule that attacks P^G^ (W1) is a key determinant of the hydrolysis efficiency. The activity decreases with distance. In the pre-hydrolysis structure (AMPPNP-bound species), His161 is flipped. Owing to the rearrangement of water molecules induced by the flipping, W1 in the active site was positioned near 3.2 Å from P^G^. W1 is deprotonated *via* a helping water molecule (W2) that forms a hydrogen bond with W1, thereby enabling the nucleophilic attack on the γ-phosphate. The proton abstracted from W1 is transiently transferred to W2 and finally delivered to one of the oxygen atoms of P_i_ which is cleaved from ATP. Thus, the configuration of W1, W2, and the P_i_ oxygen, which constitutes a proton transfer ring, is critical for the catalytic efficiency of the hydrolysis reaction. In contrast, in the post-hydrolysis structure (ADP-P_i_-bound species), P_i_ was maintained stably in the active site by intimate interactions with the P1/P2 loop residues and by a stable, short (2.5 Å) hydrogen bond with the oxygen atom of ADP. This accounts for the extreme stability of the ADP-P_i_ state in F-actin, i.e., the slow release of γ-phosphate from the filament.

The Pro-rich loop (residues 108–112) near the active site, including His161 and Gln137, is located at the interface where the domain shift associated with the G- to F-actin transition occurs. The loop is considered to be one of the regions that regulate ATP hydrolysis and P_i_ release (Wriggers and Schulten, 1999; Murakami et al., 2010; Kudryashov and Reisler, 2013). The aim of this study was to investigate the relationship between the flipping of His161, the Pro-rich loop, and ATP hydrolysis activity in F-form actin. To this end, we used three types of previously studied cardiac muscle-actin mutants (Iwasa et al., 2008; Iwasa et al., 2012): A108G (substitution of Gly for Ala108), P109A (Ala for Pro109), and Q137A (Ala for Gln137; Gln137 is hydrogen-bonded with W1). These mutants polymerize into filaments similar to wild-type actin (WT), which were examined using a conventional electron microscope. The ATPase activities of A108G and P109A, which were estimated from the rate of P_i_ release, were similar to that of the wild type (Iwasa et al., 2012). In contrast, the P_i_ release was significantly delayed from Q137A due to the low activity of ATP hydrolysis (Iwasa et al., 2008). The biochemical characteristics of the mutant actins are summarized in Supplementary Figure S1B.

In this study, we report the structures of the active sites of these actin mutants that adopt the F-form conformation. In particular, we focused on the relationship between the Pro-rich loop and His161. We found that His161 in A108G was not flipped as in G-actin, despite the overall actin structure adopting the F-form. This indicated that His161 flips to avoid steric hindrance caused by the methyl group of Ala108 approaching upon the flattening of the actin molecules. Owing to the non-flipped His161, the water network in the active site of A108G appears to be unfavorable for hydrolysis, consistent with the partial cleavage of ATP observed in the crystal but contradicting biochemical observations. In contrast, His161 in P109A is flipped; however, the imidazole ring is rotated insufficiently by the lack of bulky Pro109, which was likely stabilized by interaction with a new water molecule that replaced the proline ring. However, the water network in the active site of P109A is almost conserved, consistent with the biochemical data showing that the mutation does not significantly affect ATPase activity. Therefore, our results support the hypothesis that the rotameric conformation of His161, which is a key determinant of the configuration of the active site water molecules that catalyze hydrolysis, is governed by the Pro-rich loop.

## 2 Results

### 2.1 Structure of wild-type cardiac muscle α-actin

Human cardiac muscle actins were expressed using a baculovirus and Sf9 insect cell system (Iwasa et al., 2008; Iwasa et al., 2012). The active site of the human cardiac muscle α-actin has the same amino acid sequence as the chicken skeletal muscle α-actin, the F-form structure of which we previously determined (Supplementary Figure S1A). They differ in only four residues in the entire sequence (positions 2, 3, 299, and 358).

The expressed wild-type human cardiac α-actin (cWT) bound to ADP was complexed with the fragmin domain 1 (F1/cWT complex) and crystallized in the presence of phosphate. The crystal belonged to the same space group (*P*2_1_2_1_2_1_) as previous crystals of the complex of wild-type chicken skeletal muscle α-actin (sWT) and F1 (Kanematsu et al., 2022) with equivalent unit-cell dimensions (Table 1). The crystal structure was solved by molecular replacement using the F1/sWT complex structure (PDB code: 7W50) and refined to a resolution of 1.50 Å (Table 1). The structure of cWT in the crystal is almost identical to that of sWT adopting the F-form (sWT ADP-P_i,_ PDB code: 7W50), with an RMSD in C^α^ positions of 0.06 Å (Supplementary Figures S2B, S2F). This indicates that the cardiac actin in the complex is classified as the F-form, which is the conformation observed in actin filaments, as expected. The interface between cWT and F1 was identical to that observed for F1/sWT complexes (Supplementary Figure S2A). Furthermore, the electron density corresponding to the C^ζ^ of methylated His73 in cWT was clearly observed, and its occupancy was unity as in sWT, demonstrating that His73 was completely methylated in both the expressed and the tissue-purified actins (Supplementary Figures S2D, S2E).

**TABLE 1:** Data collection and refinement statistics.

	WT	A108G	A108G	P109A	P109A	Q137A	Q137A
	ADP-P_i_	AMPPNP	ATP·ADP-P_i_	AMPPNP	ADP-P_i_	AMPPNP	ADP-P_i_
PDB accession code	8GSU	8GSW	8GT1	8GT2	8GT3	8GT4	8GT5
Data collection							
Space group	*P*2_1_2_1_2_1_	*P*2_1_2_1_2_1_	*P*2_1_2_1_2_1_	*P*2_1_2_1_2_1_	*P*2_1_2_1_2_1_	*P*2_1_2_1_2_1_	*P*2_1_2_1_2_1_
Cell dimensions							
*a*, *b*, *c* (Å)	57.3, 91.3, 115.3	57.0, 92.8, 116.7	57.1, 90.9, 114.7	56.8, 92.8, 116.9	57.2, 91.1, 115.1	57.0, 92.8, 116.7	57.2, 91.0, 115.0
α,β,γ (°)	90, 90, 90	90, 90, 90	90, 90, 90	90, 90, 90	90, 90, 90	90, 90, 90	90, 90, 90
Resolution (Å)	48.75–1.50 (1.56–1.50)	35.88–1.40 (1.45–1.40)	29.30–1.35 (1.40–1.35)	40.74–1.50 (1.56–1.50)	42.36–1.50 (1.56–1.50)	48.59–1.55 (1.61–1.55)	35.32–1.40 (1.45–1.40)
No. of unique reflections	95,985 (9,288)	118,303 (11,397)	130,312 (12,611)	98,945 (9,702)	96,063 (9,386)	89,720 (8,779)	117,127 (11,373)
*R*_merge_	0.091 (0.885)	0.084 (1.104)	0.085 (0.943)	0.109 (1.312)	0.083 (1.059)	0.090 (1.193)	0.078 (1.064)
*R*_meas_	0.100 (0.981)	0.090 (1.199)	0.092 (1.030)	0.118 (1.437)	0.089 (1.152)	0.097 (1.299)	0.085 (1.182)
*R*_pim_	0.041 (0.418)	0.034 (0.464)	0.035 (0.405)	0.045 (0.570)	0.034 (0.448)	0.037 (0.507)	0.035 (0.505)
*CC*_*1/2*_	0.998 (0.648)	0.998 (0.620)	0.998 (0.569)	0.997 (0.472)	0.999 (0.605)	0.998 (0.551)	0.999 (0.573)
*I*/σ*I*	13.0 (1.8)	12.5 (1.7)	11.7 (1.6)	9.9 (1.4)	15.8 (1.7)	12.5 (1.5)	12.9 (1.4)
Completeness (%)	99.0 (97.0)	97.4 (94.6)	99.3 (97.2)	99.9 (99.3)	99.6 (98.6)	99.6 (98.8)	99.5 (97.8)
Redundancy	5.6 (5.1)	7.0 (6.4)	6.6 (6.0)	6.7 (6.0)	6.8 (6.2)	6.7 (6.1)	5.7 (5.2)
Refinement							
*R*_work_/*R*_free_	0.162/0.191	0.181/0.196	0.184/0.203	0.180/0.203	0.165/0.196	0.183/0.210	0.165/0.185
RMS deviations							
Bond length (Å)	0.006	0.006	0.006	0.008	0.010	0.006	0.006
Bond angle (°)	0.88	0.92	0.98	0.99	1.18	0.87	0.94
*B*-factors							
Protein	15.6	19.9	17.0	21.2	17.6	22.5	17.2
Ligand/ion	17.9	22.9	18.4	25.4	22.4	26.2	20.8
Water	30.5	32.9	31.1	33.4	32.1	34.0	30.8
Ramachandran statistics							
Favored (%)	98.6	98.6	98.6	97.8	98.2	98.4	98.4
Allowed (%)	1.4	1.4	1.4	2.2	1.8	1.6	1.6
Outliers (%)	0	0	0	0	0	0	0
All-atom clashscore	2.3	2.3	2.5	2.8	2.3	2.6	1.8
MolProbity score	1.01	1.02	1.03	1.12	1.01	1.05	0.94

**FIGURE 1:**
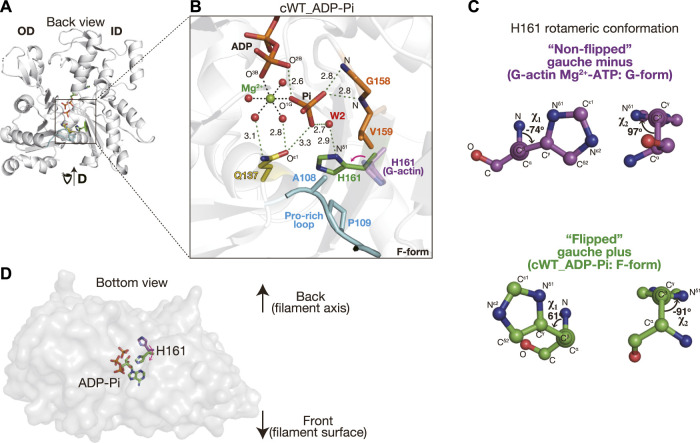
Structure of cWT_ADP-P_i_. **(A)** Structure of cWT_ADP-P_i_. The actin molecule in the crystal structure of the F1 and cWT ADP-P_i_ complex is viewed from the back side with respect to the classical presentation by Kabsch et al. (1990). The overall complex structure including the fragmin F1 domain is shown in Supplementary Figure S2A. Three residues mutated in this study (Ala108, Pro109, and Gln137), and His161 are indicated by colored sticks. Pro-rich loop (residues 108–112) is highlighted in cyan. Actin-bound nucleotide (ADP-P_i_) is depicted as sticks. **(B)** Close-up of the active site. In addition to the residues and nucleotide shown in **(A)**, Mg^2+^ (green), its coordinated water molecules (red), and a helping water molecule W2 (red) are shown as spheres. Gly158 and Val159 hydrogen bonded with P_i_ *via* the main nitrogen chain are shown in orange sticks. Key hydrogen bonds are indicated by dashed green lines with distance (Å). The distances between Mg^2+^ and the coordinated atoms (dashed black lines without digits) are 2.0–2.1 Å. His161 from a G-actin structure (4B1Y, magenta) is added to highlight the side-chain flipping associated with the G- to F-form transition. The red arrow shows the correspondence between two states and is not the rotational direction of the flipping. It should be noted that the actin conformation is indicated in the lower right corner of each panel. **(C)** Rotameric conformation of H161. The χ_1_ and χ_2_ angles of His161 in G-form and F-form actins are represented by ball-and-stick models. **(D)** His161 flipping is viewed from the bottom of the actin molecule.

In the crystal, cWT bound to ADP and P_i_, both of which were present in the crystallization drop. The ADP-P_i_-bound actin structure was also readily obtained by crystallizing sWT complexed with F1 in the presence of ADP and phosphate but has never been reported as a G-form structure. This is consistent with the considerably lower affinity of G-actin for P_i_ compared to that of F-actin (Fujiwara et al., 2007). We did not obtain a cWT structure that was bound to AMPPNP. The network of polar interactions in the active site of cWT in the ADP-P_i_ state (cWT_ADP-P_i_) (Figures 1A, B) was identical to that of sWT in the ADP-P_i_ state (sWT_ADP-P_i_) (Supplementary Figure S2G). For example, P_i_ is surrounded by O^2B^ and O^3B^ (ADP), O^ε1^ (Gln137), N (Gly158 and Val159), Mg^2+^, and several water molecules. The distance between O^3B^ (ADP) and O^1G^ (P_i_) is 2.6 Å, which is comparable to the distance observed in sWT_ADP-P_i_ (2.5 Å), confirming that a short hydrogen bond formed between the nucleotide and the cleaved P_i_ stabilized the ADP-P_i_ state (Kanematsu et al., 2022). Furthermore, W2 of the coordinating water molecules was hydrogen-bonded to N^δ1^ of His161. The rotamer conformation of histidine is expressed by two χ angles (Figure 1C): χ_1_ is the dihedral angle of N-C^α^-C^β^-C^γ^, which represents the direction of the imidazole ring. χ_2_ is the dihedral angle of C^α^-C^β^-C^γ^-Ν^δ1^ and represents the degree of rotation of the imidazole ring. In G-actin, the χ_1_ angle of His161 is usually distributed around −60°; χ_1_ = −74° and χ_2_ = 97° in an Mg^2+^-ATP G-actin structure (PDB code: 4B1Y) (Figure 1C) (Mouilleron et al., 2012). We refer to His161 in this gauche-minus conformation as non-flipped. In contrast, His161 in cWT_ADP-P_i_ adopted the gauche-plus, flipped conformation (χ_1_ = 61° and χ_2_ = −91°) (Figure 1C), as observed in sWT_ADP-P_i_ (χ_1_ = 61° and χ_2_ = −91°). Thus, the flipping of His161, from pointing to the back side to the front side of the actin (Figure 1D), appeared to be caused by a conformational change associated with the G- to F-form transition. Consequently, the observed structural similarities between cWT_ADP-P_i_ and sWT_ADP-P_i_ can safely confirm that the ATP hydrolysis mechanism proposed on the basis of crystal structures of skeletal muscle α-actin is applicable to cardiac muscle α-actin and, therefore, testable.

### 2.2 Structure of cardiac muscle α-actin mutants

To clarify the role of the Pro-rich loop in ATP hydrolysis in the F-form actin, we prepared cardiac muscle actin mutants, A108G, P109A, and Q137A. Complexes of the actin mutants and F1 were crystallized, and their structures were determined to a resolution of 1.35–1.55 Å (Table 1). For each mutant, the pre-hydrolysis state of AMPPNP-bound actin was crystallized. In addition, to obtain the post-hydrolysis ADP-P_i_ state structure, ATP-bound actin (for A108G and P109A) and ADP-bound actin (Q137A) were crystallized in the presence of phosphate (i.e., the Q137A structure does not allow us to judge whether the hydrolysis occurred in the crystal). The conformation of the actin mutants was highly similar to that of skeletal actin in the F-form (PDB code: 7W50), with an RMSD in C^α^ positions of < 0.4 Å (Supplementary Figure S2C). Therefore, actin mutants in these complexes were classified as the F-form. Although the three mutations did not affect the main-chain structure, including the Pro-rich loop (Supplementary Figure S2C), they induced changes in the water network of the active site due to the conformational changes in His161. In the following section, we describe the active site structure in actin mutants.

#### 2.2.1 Active site of A108G_AMPPNP

**FIGURE 2:**
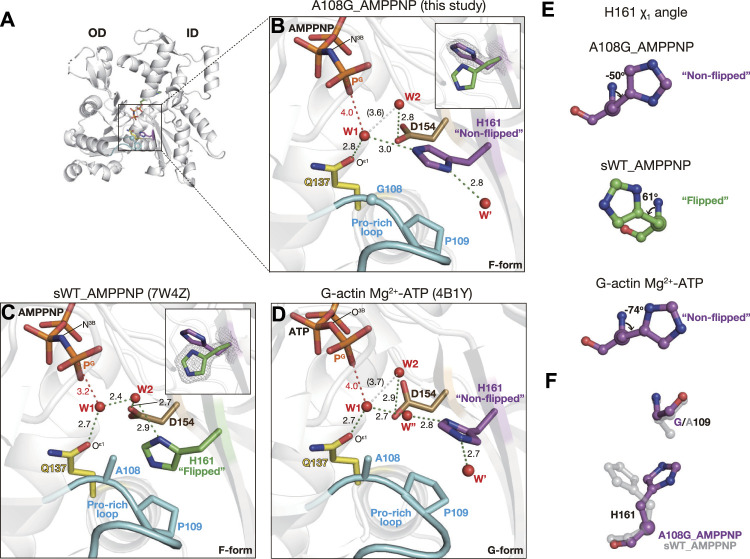
Structure of A108G_AMPPNP. **(A)** Overall structure of A108G_AMPPNP (back view). **(B–D)** Close-up views of the active site of A108G_AMPPMP [this study: **(B)**], sWT_AMPPNP [PDB code: 7W4Z, **(C)**], and Mg^2+^-ATP G-actin [PDB code: 4B1Y, **(D)**]. Nucleotides, key residues, and water molecules are shown. Red dotted lines are the distance between the gamma phosphorous atom (P^G^) of ATP/AMPPNP and putative lytic water molecules (W1). Green dotted lines represent the hydrogen bonds. White dotted lines indicate W1 and W2 that are too far apart to form a hydrogen bond. Insets in B and C show His161 in both flipped and non-flipped rotamer conformations, along with the omit map (contoured at 4σ), to specify the selected conformation in each structure. **(E)** χ_1_ angle of H161 in A108G_AMPPNP (top), sWT_AMPPNP (middle), and Mg^2+^-ATP G-actin (bottom). **(F)** Relation between His161 and the 109 position residue in A108G_AMPPNP (purple) and sWT_AMPPNP (transparent gray). A108G_AMPPNP His161 flips to avoid the methyl group of Ala109.

There are two key water molecules for ATP hydrolysis, W1 and W2, in the active site of actin. In sWT_AMPPNP (PDB code: 7W4Z, Figure 2C), W1 that is hydrogen-bonded to both O^ε1^ (Gln137) and W2 is in a position favorable for attacking P^G^ of ATP (P^G^-W1 distance = 3.2 Å; N^3B^-P^G^-W1 angle = 161°). The hydrogen bond with W1 allows W2 to help the hydrolysis reaction by deprotonating W1 and relaying a proton from W1 to P_i_ (Kanematsu et al., 2022).

In the active site of A108G_AMPPNP, most notably, the side chain of His161 was not flipped (χ_1_ = −50° and χ_2_ = −89°, Figures 2A, B, E) as in the G-form actin (χ_1_ = −74° and χ_2_ = 97° for PDB code: 4B1Y) (Mouilleron et al., 2012) (Figures 2D, E). The non-flipped His161 affects the positions of W1 and W2 (Figure 2B). W1 forms hydrogen bonds with both Gln137 and non-flipped His161 (Supplementary Figure S4B) but not with W2. As a result, while maintaining a near in-line position (167°), W1 is located far from both P^G^ (4.0 Å) and W2 (3.6 Å). Thus, the configuration of the two key water molecules in the active site suggests that the ATP hydrolysis activity of A108G is low even when the overall conformation adopts the F-form. This interpretation is supported by our A108G_ATP·ADP-P_i_ structure.

#### 2.2.2 Active site of A108G_ATP·ADP-P_i_

Actin complexed with F1 completely hydrolyzes ATP during the crystal growth (Kanematsu et al., 2022), and the refined structures of F-form complexes grown from ATP-bound WT contained only ADP and P_i_, which were defined by discrete electron densities (Supplementary Figure S3B). In the sWT_ADP-P_i_ or cWT_ADP-P_i_ structures, the attacking water W1 disappeared as it became the fourth oxygen of P_i_ upon hydrolysis (Figure 1B)

**FIGURE 3:**
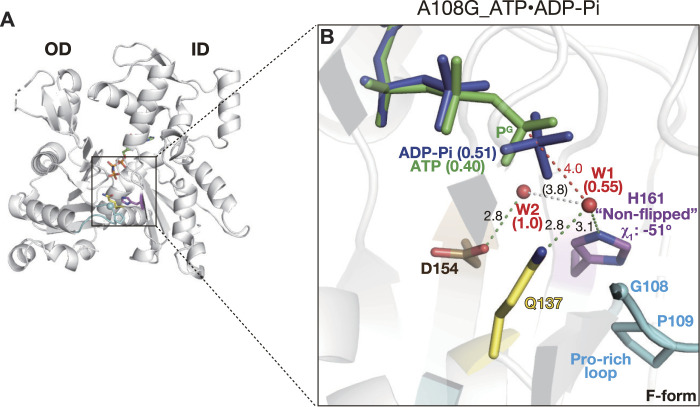
Structure of A108G_ATP·ADP-P_i_. **(A)** Overall structure of A108G_ATP·ADP-P_i_ (back view). **(B)** Close-up view of the active site. This mutant contains both ADP-P_i_ (blue) and ATP (green) in the active site, consistent with a partial hydrolysis reaction that occurred during the crystallization process. W1 has partial occupancy and is absent in the post-hydrolyzed “ATP-Pi state” conformer. The occupancy of nucleotides and W1/W2 is indicated in parentheses.

In the crystal of the F1/A108G complex prepared from ATP-bound A108G in the presence of ATP, His161 also adopted a non-flipped conformation (χ_1_ = −51° and χ_2_ = −89°, Figures 3A, B). Interestingly, in the active site, the electron density corresponding to the nucleotide was continuous, despite the high resolution of the structure at 1.35 Å (Supplementary Figure S3D). The density could best be interpreted by the concomitant placement of ADP-P_i_ (occupancy: 0.51) with ATP (0.40), demonstrating that A108G incompletely hydrolyzed bound ATP in the crystal (Figure 3B). Therefore, we refer to this structure as A108G_ATP·ADP-P_i_. Consistent with this view, in A108G_ATP·ADP-P_i_, the attacking water W1 remained partially (0.55) 4.0 Å away from P^G^ as in A108G_AMPPNP (Figure 3B). The two active site structures indicate that A108G is expected to have a lower activity of ATP hydrolysis, at least weaker than that of P109A; the P109A structure obtained by the same procedure contains only ADP and P_i_ in the active site.

#### 2.2.3 Pro-rich loop restricts the conformation of His161

**FIGURE 4:**
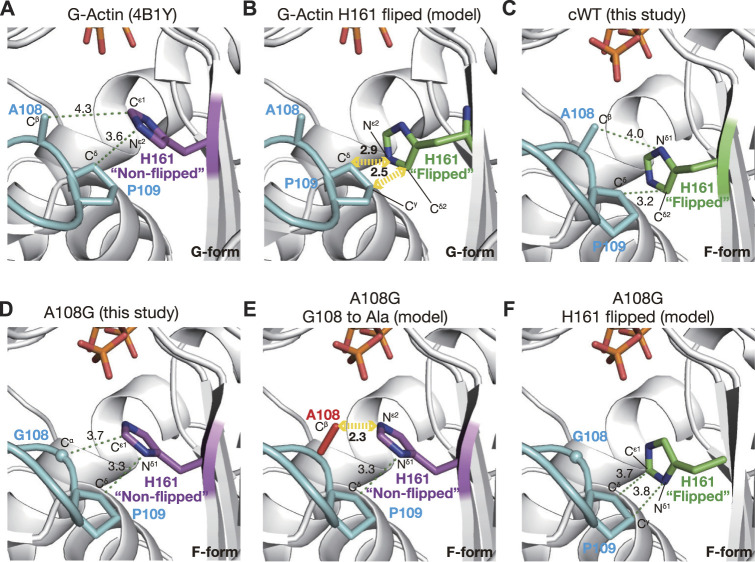
Mechanism of H161 flipping. The panels show the relationship between the Pro-rich loop and the rotameric conformations of His161 in crystal and model structures. **(A)** In a G-form actin (4B1Y), His161 adopts the non-flipped conformation. **(B)** Model structure in which His161 of 4B1Y is replaced by the flipped His161 from cWT_ADP-P_i_ demonstrates that His161 flipping is unfavorable in G-actin due to a predicted steric clash with Pro109. **(C)** In cWT_ADP-P_i_, His161 in the flipped conformation is compatible with the Pro-rich loop. **(D)** In A108G_ADP-P_i_, due to the absence of the methyl group of Ala108, His161 remains non-flipped even in the F-form actin. **(E)** Model structure based on A108G_ADP-P_i_ with the Gly108 reverted to Ala (red) demonstrates that His161 must flip its side chain in the F-form actin to avoid a steric clash with the C^β^ of Ala108. **(F)** Model structure based on A108G_ADP-P_i_ with the original His161 replaced by the flipped version shows that both His161 conformations are allowed in this mutant actin.

The conformation of His161 appeared to be associated with a short Pro-rich loop, including Pro109 and Pro112; the preceding residues 103–107 and subsequent ones 113–122 form strand and helix, respectively (Kabsch et al., 1990). Ala108 and Pro109 would behave as a rigid structure together with the preceding strand. This loop is in polar contact with surrounding residues, such as Gly74, Glu107, Arg177, and fragmin Asn13, in the crystal and has a stable conformation. Based on the geometry of the active site in A108G, the flipping of the side chain of His161 appears to be sterically caused by a shift in the methyl group of Ala108 associated with the flattening of the actin molecule (Figure 2F). To test the hypothesis as to the flipping of His161, we measured the distances between His161 and Ala108/Pro109 for the G-form, F-form, and several models. In the G-form, non-flipped His161 showed no steric clash against Ala108 and Pro109 (Figure 4A). However, replacement by flipped His161 from the F-form causes a steric clash of the flipped His161 with the side chain of Pro109; N^ε2^ (His161)–C^δ^ (Pro109) is 2.9 Å and C^δ2^ (His161)–C^γ^ (Pro109) is 2.5 Å (Figure 4B). In the F-form WT actin, flipped His161 does not collide with any residues (Figure 4C). In contrast, in the F-form A108G, non-flipped His161 has no steric clash (Figure 4D), while a replacement by the original Ala at 108 causes a steric clash between non-flipped His161 and an additional methyl group (Figure 4E). For instance, N^ε2^ (His161)–C^β^ (Ala108) is 2.3 Å. Replacement by flipped His161 in the F-form induced no steric clash (Figure 4F). This simple analysis supports the aforementioned hypothesis and shows that non-flipped His161 is favorable in the G-form, whereas flipped His161 is favorable in the F-form; in addition, A108G allows both flipped and non-flipped His161.

**FIGURE 5:**
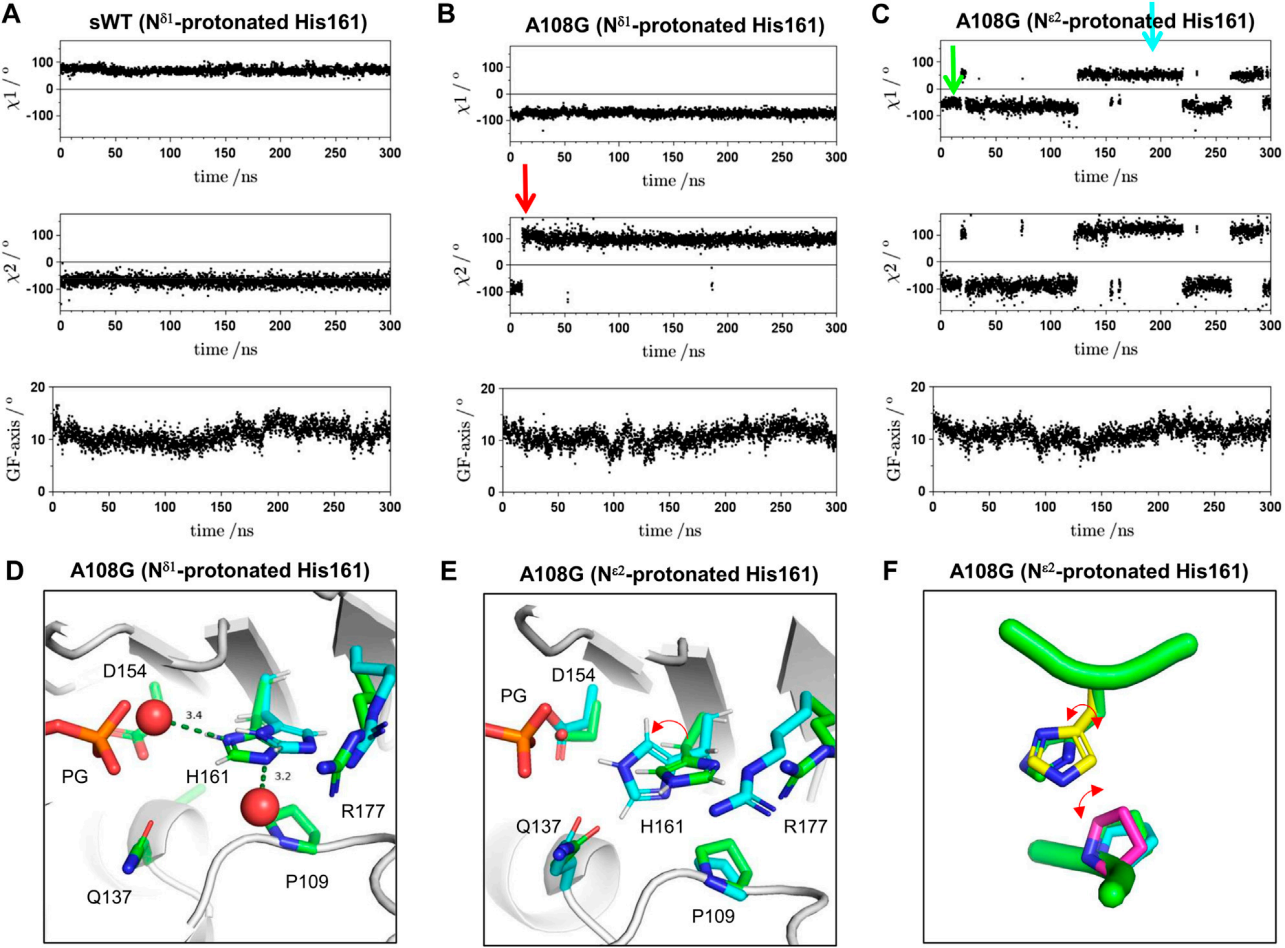
Time course for χ_1_ angle, χ_2_ angle of His161, and GF-axis value during MD simulation. **(A)** Typical trace for MD simulation on the F1/sWT complex. The GF-axis value expresses the degree of F-form in global molecule conformation and corresponds to the angle of flattering; the mean angle values of F-form and G-form are 15° and 4.5°, respectively (Oda et al., 2019). Positive and negative χ_1_ angle values indicate gauche-plus, flipped confirmation and gauche-minus, non-flipped confirmation, respectively. χ_1_ angle is ∼69° (flipped conformation). χ_2_ angle expresses the rotation of the imidazole ring of His161 and is maintained at ∼−71°. **(B)** Typical trace for MD simulation on F1/A108G complex with N^δ1^-protonated His161 (δ-tautomer). The simulation showed the imidazole ring inverted immediately after the initiation of the MD simulation (red arrow) and then maintained the upside-down orientation; χ_2_ angle is ∼−87° for 1–10 ns and is ∼96° for 20–300 ns. However, the χ_1_ angle is kept to ∼−73° (non-flipped conformation). **(C)** Typical trace for MD simulation on the F1/A108G complex with N^ε2^-protonated His161 (ε-tautomer). The χ_1_ angle is ∼−63° at 20–120 ns/260–290 ns (non-flipped conformation) and ∼43° at 120–220 ns (flipped conformation). The χ_2_ angle in the flipped conformation is ∼105° (upside-down), whose value is different from that in the F-form of WT. **(D)** Active site of A108G in the F1/A108G complex with N^δ1^-protonated His161 (δ-tautomer). Crystal structure refined at upside-down H161 (green) and MD structure after 300 ns (cyan). The two structures were pair-fitted by the main chain atoms of Gly108, Pro109, Gln137, Asp154, His161, and Arg177. **(E)** Snapshots of MD simulation on the F1/A108G complex with N^ε2^-protonated His161 (ε-tautomer) at 10 ns (green arrow in panel C) and 180 ns (cyan arrow in panel C). The two structures were pair-fitted by the main chain atom of Gly108, Pro109, Gln137, Asp154, His161, and Arg177. During the MD simulation, the flexible side chain of Arg177 frequently dissociated from the binding site. The side chain of Arg177 appeared occasionally to restrict the ring fluctuation of His161. The red arrow shows the correspondence between two states and is not the rotational direction of the flipping. **(F)** Conformations of Pro109 and His161 observed in the MD simulation on A108G (ε-tautomer). Side chains of Pro109 (cyan: ϕ = −65° and φ = 143°, magenta: ϕ = −83° and φ = 175°, where ϕ and φ are the dihedral angles of G108) were drawn on the crystal structure of A108G (A108G_AMPPNP, green: ϕ = −75° and φ = 153°). The structures were aligned using the C^α^s of residues 108–110 and 160–162. The side chains of Pro109 and His161 in the crystal do not collide with each other. However, when the side chain of His161, χ_2_, inverts in the non-flipped conformation (yellow; χ_1_ = −77.8° and χ_2_ = 83.4°), which was very rarely observed in the MD simulations, the two side chains (yellow one and magenta one) collide.

To confirm the insight obtained, 300 ns MD simulations were performed on isolated complex structures of F1/A108G or F1/sWT with ATP and crystal water molecules in the active sites, from which the changes in the χ_1_ and χ_2_ angles of His161 were measured. The GF-axis values in the bottom graphs of Figures 5A–C show the degree of flattening, which corresponds to the rotational angle between the inner (ID) and outer domains (OD) of actin; the standard values of the F-form and G-form are 15° and 4.5°, respectively (Oda et al., 2019). The GF-axis values were maintained at ∼11° for both WT and A108G regardless of the protonation state of His161, indicating that the F-form was maintained during the MD simulation, although there was a slight trend from the F-form to the G-form transition (Figures 5A–C). During the MD simulation of the F1/sWT complex with N^δ1^-protonated His161 (δ-tautomer) (Kanematsu et al., 2022), His161 remained in the flipped conformation; χ_1_ was kept at ∼69° (Figure 5A).

Since the pKa of the side chain of histidine is approximately 6, histidine is considered neutral at physiological pH. Neutral histidine could take two tautomeric states, protonated N^δ1^ or protonated N^ε2^, but crystallography cannot determine both the protonation states and the rotamers of the imidazole ring without ambiguity (Kim et al., 2013). On F1/A108G complexes, MD simulations were performed for each N^δ1^- or N^ε2^-protonated state of His161. In the case of N^δ1^-protonated His161 (δ-tautomer; Figure 5B), the imidazole ring of His161 was inverted (χ_2_; from ∼ −87° to ∼ 96°) promptly after initiation of MD simulation (red arrow in Figure 5B), and the upside-down orientation was maintained. On the other hand, the non-flipped H161 orientation was kept (χ_1_ ∼ 73°) throughout. This suggested that the upside-down ring orientation was favorable for N^δ1^-protonated His161. To investigate whether the upside-down orientation of the imidazole ring is proper in the crystal, we turned over the imidazole ring and refined the model (χ_1_ = −55° and χ_2_ = 100°). The ring, however, did not allow it to form stable bonds with the surrounding water molecules in the crystal (Figure 5D, Supplementary Video S1). This suggested that the upside-down orientation was not adopted in the crystal. Furthermore, MD simulation showed that the ring shifted by ∼0.9 Å toward the side chain of Arg177 from the crystal position (Figure 5D).

On the other hand, in the case of N^ε2^-protonated His161 (ε-tautomer), the ring formed stable hydrogen bonds with the surrounding water molecules (Supplementary Video S2), and the shift of the ring was small (Figure 5E; ∼ 0.5 Å). This suggested that non-flipped His161 in A108G would adopt ε-tautomer. During four runs of the simulation of the F1/A108G complex with His161 of ε-tautomer (Figure 5C), a transition was observed between non-flipped H161 (χ_1_ ∼ −64°) and flipped His161 (χ_1_ ∼ 46°). Figure 5E shows snapshots of the active site at 10 ns (green: non-flipped H161) and 180 ns (cyan: flipped H161). Hence, the result supported the previously mentioned insight that A108G allows both flipped and non-flipped His161 in the F-form.

#### 2.2.4 Active site of P109A_AMPPNP

**FIGURE 6:**
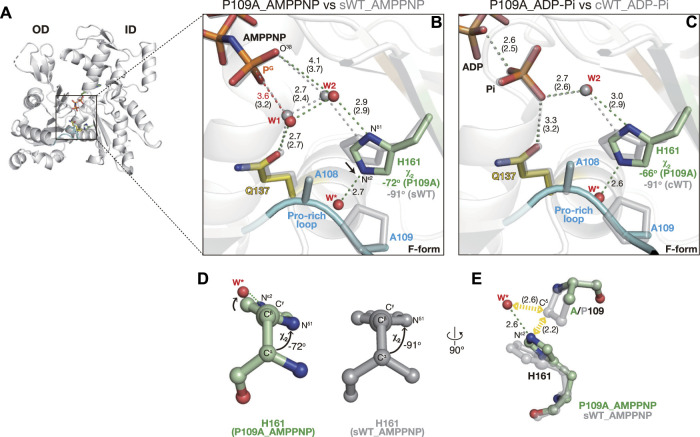
Structures of P109A. **(A)** Overall structure of P109A_AMPPNP (back view). **(B–C)** Close-up views of the active site of P109A_AMPPNP **(B)** and P109A_ADP-P_i_ **(C)**. For comparison, nucleotides, key residues, and water molecules from sWT_AMPPNP **(B)** or cWT_ADP-P_i_ **(C)** are shown by transparent gray sticks/spheres. W* is a water molecule hydrogen bonded with the N^ε2^ of His161 present only in the mutants. Distances (Å) between key atoms are indicated with corresponding distances in the reference structures in parentheses. **(D)** χ_2_ angle of His161 in P109A_AMPPNP (left) and sWT_AMPPNP (right). **(E)** Mechanism of His161 tilting. The loss of the proline ring allows (1) the approaching of His161 toward the Pro-rich loop, and (2) the entering of a water molecule (W*). His161 in a tilted conformation is stabilized by a hydrogen bond between N^ε2^ and W*.

In P109A AMPPNP, polar interactions in the active site were essentially similar to those in sWT_AMPPNP, and His161 was flipped (χ_1_ = 67° cf. χ_1_ = 61° for sWT AMPPNP) (Figures 6A, B). According to the backbone-dependent side-chain library (Shapovalov and Dunbrack, 2011), the χ_2_ angle is distributed around −82° or +89° for a histidine side chain with χ_1_ ∼+60°, ϕ = −140°, and φ = 150°, which are comparable to the dihedral angles of His161 (φ = −147° and φ = 155° cf. ϕ = −135° and φ = 150° for sWT AMPPNP). The χ_2_ angle of His161 in sWT_AMPPNP was −91°. N^δ1^ forms a hydrogen bond with W2 and its imidazole contacts with Pro109 by weak CH–π interaction (Brandl et al., 2001), which likely restricts the orientation of the His ring (Figure 2C). In contrast, the χ_2_ angle of His161 in P109A AMPPNP was −72° (Figure 6B). In addition to the N^δ1^–W2 bond, N^ε2^ of His161 was hydrogen-bonded with a water molecule (W* in Figure 6B; Supplementary Figure S4D) that replaced the proline ring. The less favorable His161 ring conformation is likely stabilized by the N^ε2^–W* bond as W* is in a position off the ideal ring plane (Figures 6D, E).

The ∼20° less rotation of the His161 imidazole ring resulted in subtle shifts in the two key water molecules. W1 is slightly further away from the P^G^ of AMPPNP, from 3.2 Å in sWT_AMPPNP to 3.6 Å (Figure 6B). W2 is also slightly separated from O^3G^ of AMPPNP, from 3.7 Å to 4.1 Å. In addition, the distance between W1 and W2 is slightly longer, from 2.4 Å to 2.7 Å, likely explaining the increment of the W1 occupancy from 0.68 to 0.89. It is noteworthy that the too-short contact between W1 and W2 disappeared, likely due to the incomplete rotation of His161, which permitted the shift of W2 against W1. The very short contact also disappeared after the geometry optimization of sWT_AMPPNP, which was used as a reactant structure in the ATP hydrolysis analysis (Kanematsu et al., 2022). The optimization shifted the distances of O^3G^–W2 and W1–W2 from 3.7 Å to 4.1 Å and from 2.4 Å to 2.7 Å, respectively. These geometries in the hydrolysis-ready structure were comparable to those of P109A_AMPPNP, suggesting that the proton transfer process through W1–W2-O^3G^ properly proceeds in P109A. Although the longer distance of P^G^-W1 would be unfavorable for reactivity, the increasing occupancy of W1 might compensate for the opportunity for attacking P^G^. In summary, although the efficiency cannot be determined, P109A is expected to have ATP hydrolysis activity, which is supported by the P109A_ADP-P_i_ structure in the following section. Finally, *Dictyostelium* P109A in complex with human gelsolin segment 1 (PDB code: 3A5L (Murakami et al., 2010)) adopts the G-form with non-flipped His161 (χ_1_ = −77.7°), indicating that together with our F-form structures, P109A also obeys a scheme in which the side-chain flipping of His161 occurs upon G- to F-form transition.

#### 2.2.5 Active site of P109A_ADP-P_i_

Crystallization of the F1/P109A complex in the presence of ATP yielded an F-form structure with ADP and P_i_ (no ATP) in the active site, indicative of complete hydrolysis (Figure 6C, Supplementary Figure S3F). His161 was flipped, and its imidazole ring was rotated incompletely, as in P109A_AMPPNP (χ_1_ = 64° and χ_2_ = −66°). In addition, the polar interactions in the active site of P109A_ADP-Pi (Figure 6C) were highly similar to those in cWT_ADP-P_i_ (Figure 1B), implying that the mutation had no effect on the affinity of P_i_ to actin.

#### 2.2.6 Active site of Q137A_AMPPNP

**FIGURE 7:**
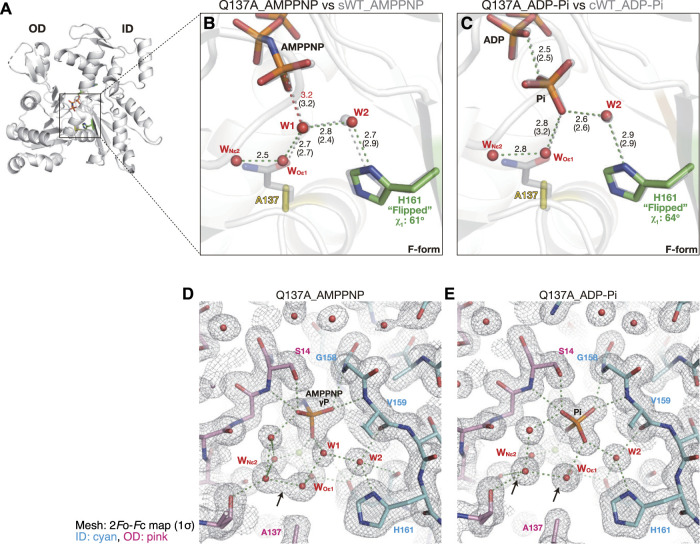
Structures of Q137A. **(A)** Overall structure of Q137A_AMPPNP (back view). **(B–C)** Close-up views of the active site of Q137A_AMPPNP **(B)** and Q137A_ADP-P_i_ **(C)**. For comparison, nucleotides, key residues, and water molecules from sWT_AMPPNP **(B)** or cWT_ADP-P_i_ **(C)** are shown by transparent gray sticks/spheres. W_Oε1_ and W_Nε2_ are water molecules replacing O^ε1^ and N^ε2^ of Q137 in the wild-type actins, respectively. Distances (Å) between key atoms are indicated (corresponding distances in the reference structure are in parentheses). **(D–E)** 2*F*o-*F*c electron density map contoured at 1σ with Pγ/P_i_ in the center of the panel. The ID and OD of actin are colored cyan and pink, respectively. In Q137A_AMPPNP **(D)**, W_Oε1_ and W_Nε2_ are only 2.5 Å apart and in a continuous density (arrow). In Q137A_ADP-P_i_ **(E)**, the two water molecules are separated at 2.8 Å with distinct densities (arrows), likely due to the inversion of the P_i_ configuration that leads to a rearrangement of the surrounding water molecules.

The G- to F-form transition brings the side chain of Gln137 closer to the nucleotide (Merino et al., 2018; Chou and Pollard, 2019). The mutation of Gln137 to Ala had no effect on the C^α^ position of residue 137 (Figures 7A, B), demonstrating that the shift was induced by a conformational change occurring at the main chain level. In the crystal of sWT_AMPPNP, the side chain of Gln137 was hydrogen-bonded to both W1 and Mg^2+^-coordinated water molecules. Despite the absence of the side chain of Gln137, the polar interactions in the active site of Q137A_AMPPNP were highly similar to those of sWT_AMPPNP (Figure 7B), and His161 was in a flipped conformation (χ_1_ = 61° and χ_2_ = −84°). The lack of the side chain was compensated for by two water molecules, W_Oε1_ and W_Nε2_, which occupied the positions corresponding to O^ε1^ and N^ε2^ (Gln137). As a result, W1 loses its direct interactions with actin residues (Figure 7B, Supplementary Figure S4C). In addition, W_Oε1_ does not bind directly with actin residues. The distance between W1 and P^G^ is 3.2 Å, and it is the same as that for sWT_AMPPNP, while the distance between W1 and W2 is slightly longer, from 2.4 Å to 2.8 Å. Judging from the configuration of W1 and W2, Q137A appears to have ATP hydrolysis activity comparable to the wild-type actin. However, this expectation is inconsistent with biochemical data, as discussed in the “*Discussion*” section.

#### 2.2.7 Active site of Q137A_ADP-P_i_

The crystal structure of the F1/Q137A complex, obtained in the presence of ADP and phosphate, showed clear electron densities assignable to ADP and P_i_ in the active site of F-form actin (Supplementary Figure S3H). The polar interactions in the active site of Q137A_ADP-P_i_ were highly similar to those of cWT_ADP-P_i_, and His161 was flipped (χ_1_ = 64° and χ_2_ = −89°; Figure 7C). In the crystal structure of Q137A_AMPPNP, the two water molecules replacing the side-chain atoms of Gln137, W_Oε1,_ and W_Nε2_, had a continuous electron density and were separated by only 2.5 Å (indicated by an arrow in Figure 7D). Notably, the two water molecules in Q137A_ADP-P_i_ were in discrete density with a separation of 2.8 Å (Figure 7E), which is the theoretical optimum hydrogen-bond length formed between water molecules. Thus, in terms of the configuration of the water molecules in the active site, Q137A_ADP-P_i_ (post-hydrolysis structure) is less strained because of the geometric optimization elicited by P_i_ cleavage.

## 3 Discussion

In the present study, to analyze the mechanism of ATPase in actin filaments, we prepared the mutant actins, A108G, P109A, and Q137A, and determined the crystal structures of the pre-hydrolysis (AMPPNP-bound species) and post-hydrolysis (ADP-P_i_ bound species) structures in the F-form. The results are summarized in Table 2. It is well-established that the conformational change in actin from the G-form to F-form (flattening) is accompanied by a change in His161 from the non-flipped to the flipped state (Merino et al., 2018; Chou and Pollard, 2019; Kanematsu et al., 2022). As shown in Table 2, in the F-form actins, the degree of flattening and/or the bound nucleotide have little effect on the χ_1_ and χ_2_ angles of His161. Mutations in the Pro-rich loop residues affect the rotameric states of His161. We focus on changes in the local interactions and water network of the active site due to the loss of side chains (A108G, P109A) and discuss the various conformations of His161 observed in the F-form actins. It is noted that the Q137A mutation does not affect the rotameric state of His161.

**TABLE 2:** Summary of actin conformation, nucleotide state, and His161 conformation.

WT/mutant	Nucleotide	His161 χ_1_ /^o^ ^a^	His161 χ_2_ /^o^	GF-axis /^o^ ^b^	GC-axis /^o^	PDB code
sWT	AMPPNP	61	−91	14.7	−0.6	7W4Z
sWT	ADP-P_i_	61	−91	15.0	−0.7	7W50
cWT	ADP-P_i_	61	−91	15.1	−0.5	In this study
sWT	ADP	49	−91	14.8	−0.1	7W51
P109A	AMPPNP	67	−72	12.5	−0.5	In this study
P109A	ADP-P_i_	64	−66	14.9	−0.1	In this study
Q137A	AMPPNP	61	−84	13.1	−0.7	In this study
Q137A	ADP-P_i_	64	−89	15.1	−0.6	In this study
A108G	AMPPNP	−50	−89	12.9	−0.2	In this study
A108G	ATP + ADP-P_i_	−51	−89	14.5	0.02	In this study
sWT (G-form)	ATP	−74	97	5.2	−6.3	4B1Y

^a^ The χ_1_ angle of sWT_ADP deviates significantly from other WT F-form actins likely due to the double conformation of the P2-loop (residues 156–159) near His161 which perturbs water orientation.

^b^ The GF-axis values of mutant AMPPNP actins (12.5–13.1°) are smaller (i.e., twisted) than those of other F-form actins including sWT_AMPPNP (∼15°).

We found that the conformation of His161 in A108G actin was the same as that of G-actin, and His161 was not flipped despite its global conformation in the F-form. This indicates that the methyl group at residue 108 on the Pro-rich loop is critical for the flipping of His161, and the flipping appears to be sterically caused by a shift in Ala108 associated with the flattening of actin molecules (G- to F-form transition). Furthermore, the flipping of His161 is necessary for ATP hydrolysis and appears to be a trigger. On the other hand, the orientation of the imidazole ring would be fixed by interaction with shifted Pro109 (Figure 4) or by preventing excess water (W* in Figure 6B) from entering the active site owing to the bulky Pro109. In summary, the conformation of His161 in the F-form actin is governed by the Pro-rich loop.

Some predictions derived from these crystal structures are seemingly at odds with the previous biochemical data. Judging solely from the positions of W1 (attacking water) and W2 (helping water) in crystals, P109A and Q137A are expected to have an ATP hydrolysis activity similar to that of WT, while A108G is expected to have extremely low activity. In contrast, biochemical experiments have shown that the ATPase activities of A108G and P109A are similar to those of WT, whereas that of Q137A is low (Iwasa et al., 2008; Iwasa et al., 2012). Although we do not fully understand the discrepancy, in the following section, we will discuss a possible explanation for the inconsistency between the structural and biochemical data for A108G and Q137A.

First of all, we considered the ATP hydrolysis activity of A108G. The side chain of His161 in the canonical G-actin is not flipped, and the flipping is also inhibited by steric hindrance against the Pro-rich loop (Figures 4A, B); consequently, ATP bound in the active site is usually observed in the G-actin crystal obtained in the presence of ATP. In addition, in the case of A108G adopting the F-form, although the side chain of His161 was not flipped in the crystals, the flipping itself would not be inhibited by steric hindrance (Figures 4D–F) and it would occur occasionally as shown by the MD simulation (Figure 5C). This suggests that A108G in the F-form conformation can flip His161 and consequently has ATP hydrolysis activity, which is evident from the partial cleavage of ATP in the active site of the A108G crystal (Figure 3B).

The MD simulation showed no unique path of the flipping of His161 in A108G. However, flipping of His161 seems to be associated with the ring orientations and fluctuations of Pro109 and His161 that appear specifically by mutation of Ala108 to Gly (Figure 5F; Supplementary Figure S5), likely allowing the configuration of the two adjacent side chains to change in the restricted space of the active site. If this is the case, then the flipping might be facilitated by increased flexibility of the Pro-rich loop including Pro109, and accordingly, its frequency might increase the chance of triggering ATP hydrolysis. The loop in the crystal is fixed through multiple hydrogen bonds of Ala108, Leu110, and Asn111 in the Pro-rich loop with fragmin Asn13 (Kanematsu et al., 2022). On the other hand, in the filament, only Pro112 sterically contacts with Glu195–Gly197 of the diagonal actin subunit (Chou and Pollard, 2019). It is possible that the A108G subunit in the filament is more prone to hydrolysis than that in the crystal due to the greater fluctuation of the Pro-rich loop. However, further studies are needed.

Q137A appeared to have ATPase activity comparable to that of wild-type actin because the position of the attacking water molecule W1, a key determinant of the hydrolysis reaction, was unaffected by the mutation (Figure 7). However, this structural information does not agree with our previous biochemical observation that this mutation significantly slows hydrolysis (Iwasa et al., 2008). QM/MM analysis (Kanematsu et al., 2022) revealed that although Gln137 does not participate in the catalytic reaction, the side-chain oxygen/nitrogen still contributes to the reaction by maintaining W1, and the reaction intermediates at the catalytic site *via* direct or indirect hydrogen bonding (Kanematsu et al., 2022). Thus, the replacement of the Gln137 side-chain atoms by the water molecules, W_Oε1_ and W_Nε2_, may disturb the hydrolysis reaction because of the increased fluctuation of the water network in the catalytic site in which the two water molecules, W1 and W_Oε1_, do not bond directly to any actin residue. To analyze the fluctuation of water molecules in the active site, we performed 15-ns MD simulations with harmonic restrictions for the heavy atoms (Young et al., 2007). The positions of the water molecules in the active site of G-actin (PDB code: 4B1Y) were maintained during the MD simulation, and the fluctuation was small (Supplementary Video S3). In contrast, in the case of the active site of sWT, W2 was maintained, W1 moved, and W1 was occasionally exchanged (Supplementary Video S4), which may reflect the difference in occupancy between W1 (0.68) and W2 (1) in sWT_AMPPNP (Kanematsu et al., 2022). In the case of Q137A, water molecules in the cluster, including W1, W_Oε1_, and W_Nε2_, were rapidly exchanged, such that the W1 position was always occupied by one in the water cluster (Supplementary Video S5). The dynamics of the water molecules may explain the low ATPase activity of Q137A. Fluctuations in the real system, which include peptide chain dynamics, are more complicated. However, our analysis further implies that information about not only the static structure but also its dynamics is necessary to understand enzyme reactions. The dynamics of water molecules, which frequently substitute bulky amino acids for small amino acids, are essential for interpreting mutant experiments.

Finally, in this study, the effects of the mutations on ATPase activity are discussed in terms of the arrangement of the attacking water molecule W1 and helping water molecule W2. To understand the entire ATP hydrolysis process, QM/MM calculations must be performed. The system is expected to be able to perform QM/MM calculations because the structures of the pre- and post-states are available at a high resolution. Not surprisingly, the mutations introduced in the actin residues also affect filament assembly; P109A and Q137A polymerize faster than wild-type actin, whereas A108G polymerizes much slower (Iwasa et al., 2008; Iwasa et al., 2012). We did not discuss the molecular assembly in this study because the crystals contain actin molecules adopting the F-form without polymerization, and inter-subunit interfaces present in F-actin, including the D-loop mediated intra-strand interaction, are not reproduced in our structures. The three mutant actins polymerize into filaments with canonical helical parameters (Iwasa et al., 2008; Iwasa et al., 2012). Cryo-EM analysis may address how mutations in the active site propagate to the peripheral inter-subunit interface that impacts elongation.

## 4 Materials and methods

### 4.1 Protein expression and purification

Human cardiac muscle α-actins, wild-type (WT), and mutants (A108G, P109A, and Q137A), which were fused to 16 amino acid residues containing a Strep-Tag II affinity tag at their N-terminus, were prepared according to a previously described method with modifications (Iwasa et al., 2008; Iwasa et al., 2012). Briefly, recombinant actins expressed in a baculovirus-Sf9 cell system were purified by affinity chromatography using the Strep-tag, followed by gel filtration chromatography in the presence of a high concentration (1 M) of Tris to dissociate endogenous insect cofilin that bound tightly to exogenous human actin. The isolated actin-containing fractions were then concentrated and used to prepare the F1A complex. The gel-filtrated actin samples used for the crystallization of cWT_ADP-Pi and Q137A_ADP-P_i_ were further polished by one cycle of polymerization and depolymerization. The *Physarum polycephalum* fragmin F1 domain (residues 1–160), expressed using an *Escherichia coli* expression system, was purified as described previously (Takeda et al., 2020).

### 4.2 Preparation of the fragmin F1/actin complex

Fragmin F1 domain/actin complexes were prepared using a previously described method (Kanematsu et al., 2022) with modifications.

*cWT_ADP-P*_*i*_ *, Q137A_ADP-P*_*i*_: Purified WT or Q137A was mixed with F1 preincubated with 1 mM CaCl_2_ at a molar ratio of 1:1.2. Using a centrifugation concentrator (MWCO 50,000), the complexes were concentrated and buffer-changed to ADP-G-buffer (2 mM Tris-HCl pH 8.0, 0.2 mM CaCl_2_, 0.2 mM ADP, and 1 mM DTT).

*A108G_ATP·ADP-P*_*i*_, *P109A_ADP-P*_*i*_: Gel-filtrated A108G or P109A were dialyzed against ATP-G-buffer (2 mM Tris-HCl pH 8.0, 0.2 mM CaCl_2_, 0.2 mM ATP, and 1 mM DTT). Excess Tris-removed G-actins were polymerized by adding an equal volume of ADP-F-solution (0.2 M KCl, 20 mM imidazole pH 7.0, 2 mM MgCl_2_, 0.4 mM EGTA, 0.2 mM ADP, and 2 mM DTT). F-actin pellets, collected by ultracentrifugation, were resuspended in ATP-G-buffer and dialyzed against the same buffer. Depolymerized recombinant actins were complexed with the Ca^2+^-activated F1 and concentrated/buffer-exchanged to ATP-G-buffer.

*A108G_AMPPNP, P109A_AMPPNP, Q137A_AMPPNP*: Complexes containing mutant actins bound to a slowly hydrolyzed ATP analog β,γ-imidoadenosine 5′-triphosphate (AMPPNP) were prepared using the method described in the previous paragraph, using AMPPNP (0.1 mM)-G-buffer instead of ATP-G-buffer.

The actin-bound nucleotides in the F1/actin complexes used for crystallization are as follows: ATP (A108G_ADP-P_i_ and P109A_ADP-P_i_), ADP (cWT_ADP-P_i_ and Q137A_ADP-P_i_), and AMPPNP (A108G_AMPPNP, P109A_AMPPNP, and Q137A_AMPPNP).

### 4.3 Crystallization and structural determination

The protein concentration of the F1/actin complexes used for crystallization was 10 mg/mL, except for Q137A_ADP-P_i_ which was 3.8 mg/mL. Before crystallization, the nucleotide-bound cation (Ca^2+^) was exchanged for Mg^2+^ by a 5-min incubation with 0.5 mM EGTA and 10 mM MgCl_2_. The aggregates were removed by brief centrifugation. Crystals were obtained using the hanging drop vapor diffusion method at 20°C by mixing equal volumes (1 µL) of the protein solution and the reservoir solution (17%–19% (w/v) PEG3350, 0.1 M Na_2_HPO_4_, 0.1 M HEPES-NaOH pH 8.0). To facilitate crystal growth, streak seeding was performed using a seed drop containing the F1A crystals composed of F1 and chicken skeletal muscle α-actin. Crystal growth was not disturbed by the uncleaved tag sequence at the N-terminus of actin, which also had no influence on actin polymerization/depolymerization (Iwasa et al., 2008; Iwasa et al., 2012). Crystals were cryoprotected by brief soaking in mother liquor supplemented with 15% (v/v) ethylene glycol and flash-cooled in a cold nitrogen stream.

X-ray diffraction measurements were performed on beamline BL2S1 at the Aichi Synchrotron Radiation Center (Watanabe et al., 2017) with a wavelength of 1.12 Å at −183°C. Datasets collected from a single crystal were processed using XDS (Kabsch, 2010). The initial phase was obtained by molecular replacement with Molrep (Vagin and Teplyakov, 2010) using the F1/sWT complex structure (PDB code 7W50) as a search model. The isoform-specific and mutated residues, evident at this stage, were corrected using Coot (Emsley et al., 2010). The structural models were refined by iterative rounds of restrained refinement and manual inspection using Refmac5 (Murshudov et al., 1997), Phenix.refine (Afonine et al., 2012), and Coot. Because of a slight carryover from a seed drop and the relatively high resolution of the produced crystals (1.35–1.55 Å), in some structures, a trace of electron densities, which could be attributable to the wild-type chicken actin residues, was observed, although not at the level of placing them as alternative conformers. The nucleotide species in the actin active site are as follows: AMPPNP: A108G_AMPPNP, P109A_AMPPNP, and Q137A_AMPPNP (AMPPNP was not hydrolyzed during crystal growth as expected), ADP-P_i_: cWT_ADP-P_i_, P109A_ADP-P_i_, and Q137A_AMPPNP (in P109A_ADP-P_i_ starting from ATP-actin, ATP was completely hydrolyzed during crystal growth and P_i_ was incorporated from the mother liquor, while in the other two species using ADP-actin, P_i_ was incorporated into the ADP-actin crystals), and a mixture of ATP and ADP-P_i_: A108G_ATP·ADP-P_i_ (actin-bound ATP was not completely hydrolyzed, so the pre- and post-hydrolysis states were coexisting). The data collection and refinement statistics are summarized in Table 1. All final structure models have one copy of the F1/actin complex in the asymmetric unit and consist of fragmin residues 7–160 (142 and 143 are missing in some structures) and actin residues 5–41, 50/51–375. All the structural figures were prepared using PyMOL (Schrödinger and DeLano, 2020).

### 4.4 MD simulation

MD simulations were performed using the GROMACS package (Abraham et al., 2016). The missing regions of the initial crystal structures were added using the Modeller 9.1 package (Sali and Blundell, 1993) or other actin crystal structures such as the N-terminus region of 1ATN and the D-loop of the unpublished actin-fragmin crystal structure. AMPPNP was converted to ATP by exchanging N^3B^ with O^3B^. The initial proteins were solvated in a rectangular box with a minimum distance of 1.0 nm using the crystal water within 12 nm around AMPPNP and the water generated by GROMACS. Potassium and chloride ions (100 mM) were added for the net charge of the system. Electrostatic interactions were calculated using the particle-mesh Ewald algorithm (Essmann et al., 1995), and all bond lengths were constrained using the linear constraint solver algorithm (Hess, 2008). The temperature and pressure were 300 K and 1 atm using a v-rescale thermostat (Bussi et al., 2007) and a Parrinello–Rahman barostat (Parrinello and Rahman, 1981), respectively. After energy minimization of the system using the steep method, the system was equilibrated under constant volume and temperature for 500 ps with position restraints for the heavy atoms of the protein, Mg^2+^, and ligand, followed by equilibration for 500 ps under constant pressure and temperature. Subsequently, MD productions were performed. In the 300-ns MD simulation, CHARMM36 (July 2021) and TIP3p force fields (Mackerell et al., 2004), actin–fragmin complexes as the initial structure, and FUJITSU PRIMERGY CX2570 M5 computer (Nagoya University Information and Communications) were used. In the 15-ns MD simulations with harmonic restraints of 1,000 kJ/mm^2^ for heavy atoms of protein, Mg^2+^ and ATP, CHARMM27/CMAP, and TIP4 force fields, isolated actin as an initial structure, and a workstation with a GPU (Tegra Co., Hamamatsu) were used. The traces were analyzed using GROMACS, Scilab (Group E, 2023), and PyMOL (Schrödinger and DeLano, 2020).

## Data Availability

The datasets presented in this study can be found in online repositories. The names of the repository/repositories and accession number(s) can be found at: https://www.rcsb.org/structure/8GSU, https://www.rcsb.org/structure/8GSW, https://www.rcsb.org/structure/8GT1, https://www.rcsb.org/structure/8GT2, https://www.rcsb.org/structure/8GT3, https://www.rcsb.org/structure/8GT4, https://www.rcsb.org/structure/8GT5.
