# Supplementary material for: Mutagenic analysis of actin reveals the mechanism of His161 flipping that triggers ATP hydrolysis
Source: Front Cell Dev Biol. 2023 Mar 16;11:1105460. doi: 10.3389/fcell.2023.1105460 (PMC10062479; doi:10.3389/fcell.2023.1105460)
Supplement: Supplementary file 4 [file DataSheet1.pdf]

Supplementary Material

1 Supplementary Figures

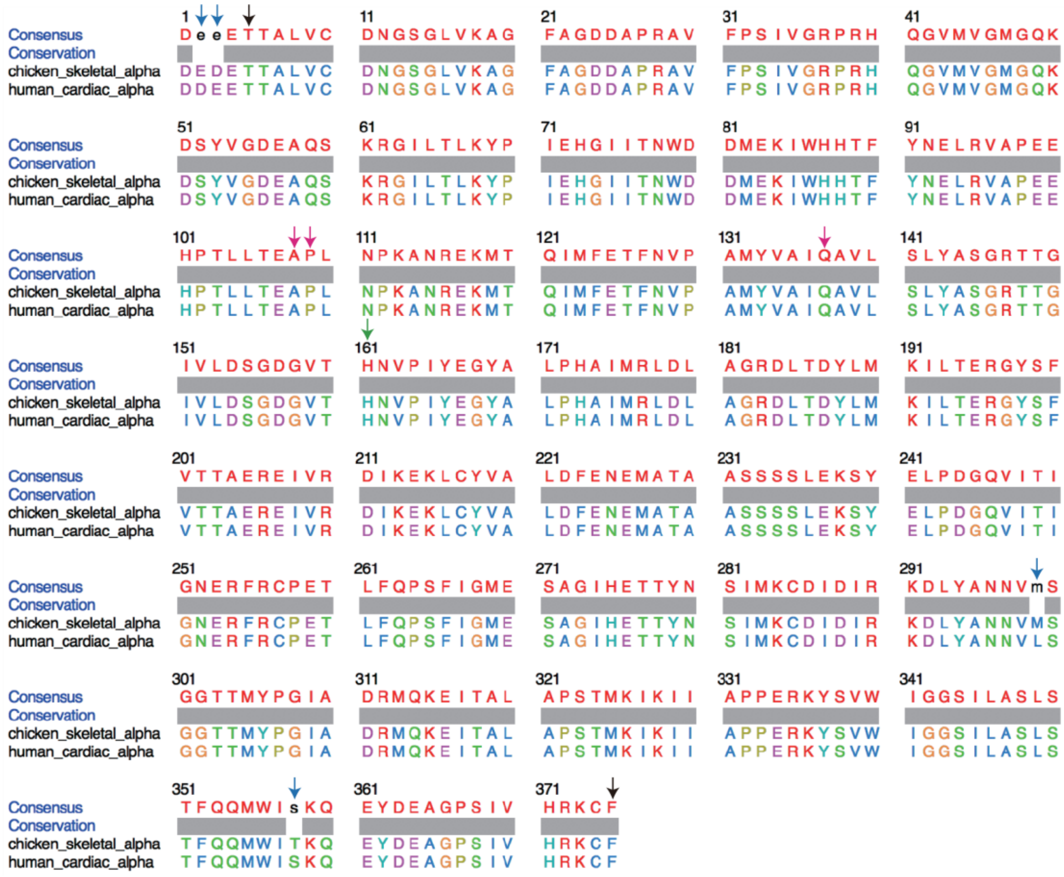

B

	Polymerization	ATPase
A108G	slow	similar
P109A	fast	similar
Q137A	fast	slow

Supplementary Figure S1. Comparison between human cardiac α-actin with chicken skeletal α-actin

(A) Sequence alignment. The amino acid sequence of human cardiac α-actin (UniProt P68032) used in this study was aligned with that of chicken skeletal α-actin (UniProt P68139) we previously used to obtain the F-form actin structures (1). The first two residues (Met-Cys) cleaved by post-translational modifications are omitted. Arrows indicate follows, Blue: residues different between the two actin isoforms (Asp2, Glu3, Lue299, Ser358), Pink: residues mutated in this study (Ala108, Pro109, Gln137), Green: a key histidine flipped by G- to F-form transition (His161), Black: The N- and C-terminus residues observed in the cWT_ADP-Pi structure (Thr5, Phe375).

(B) Biochemical characteristics of the mutant actins. The effect of three mutations (A108G, P109A, and Q137A) on the polymerization and ATPase activity compared to the wild type actin, examined in (2, 3), are summarized.

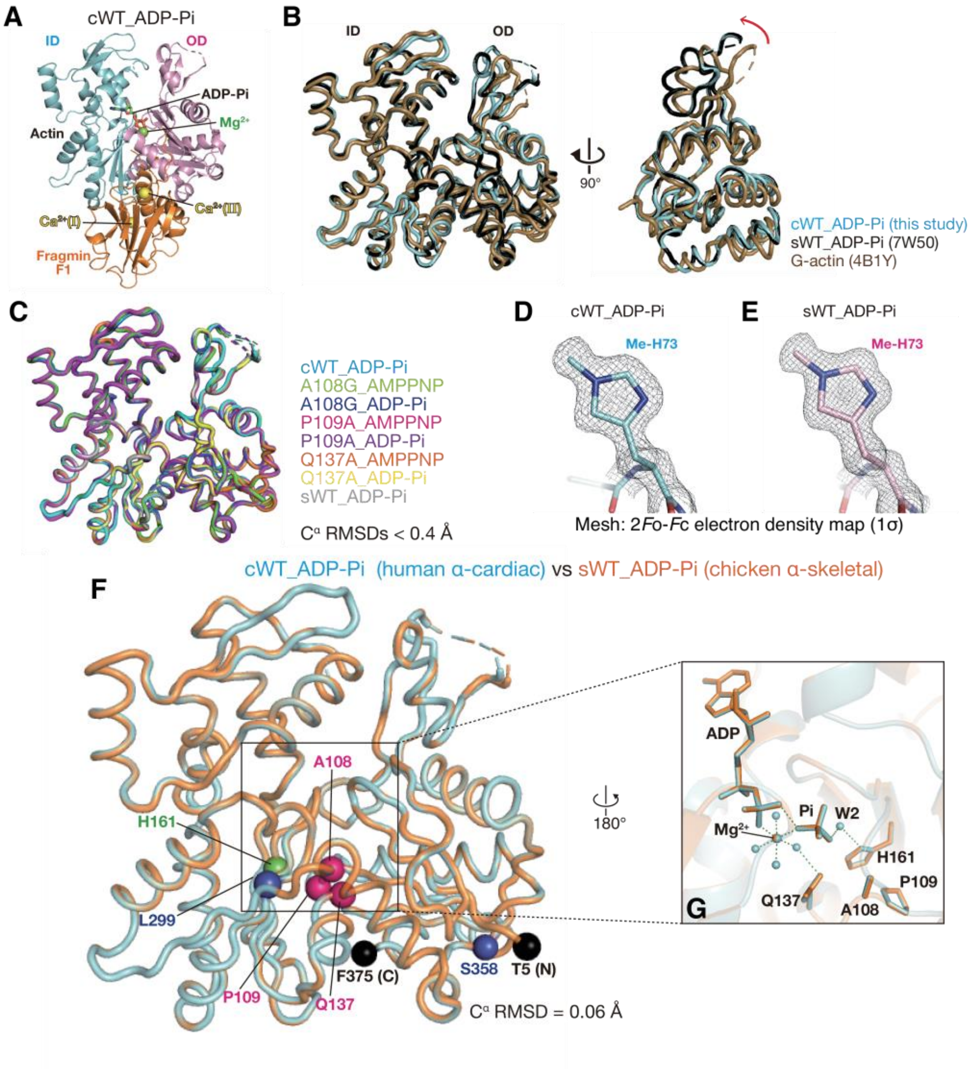

Supplementary Figure S2. Structure of cWT_ADP-Pi

(A) Overall structure of cWT_ADP-Pi in complex with fragmin F1. Actin (ID: cyan, OD: pink) and fragmin F1 domain (orange) are shown in the cartoon models. Actin-bound nucleotide (ADP-Pi) and cation (Mg^{2+}: green) are depicted as sticks and a sphere, respectively. Calcium ions coordinated at the conserved binding sites (types I and II) (4) are shown as yellow spheres.

(B) Actin conformation. Superimpositions of three actin structures, viewed from front (left) or the OD side (right), are shown. Cyan: cWT_ADP-Pi (human cardiac α, this study). Black: sWT_ADP-Pi (chicken skeletal α, PDB code:7W50). Brown: Mg^{2+}-ATP-G-actin (rabbit skeletal α, 4B1Y). A red curved arrow indicates a domain rotation from G-form to F-form.

(C) Actin structure comparison. Seven actin structures reported in this study, shown in different colors, were superimposed onto the actin molecule from sWT_ADP-Pi (7W50) (1), with C^{α} RMSDs less than 0.4 Å.

(D-E) Methylation of His73. His73 in cWT_ADP-Pi (D) or sWT_ADP-Pi (E) with 2*F*o-*F*c electron density map (contoured at 1σ) show that His73 is post-translationally methylated in both actins.**(F-G)** Structural comparison. Overall actin structures of cWT_ADP-Pi (cyan) and sWT_ADP-Pi (orange) were superimposed with a C^{α} RMSD value of 0.06 Å. (F) Key residues indicated in Fig. S1A were depicted as spheres. An enlarged view emphasizes the identity of the two structures in the active site (G).

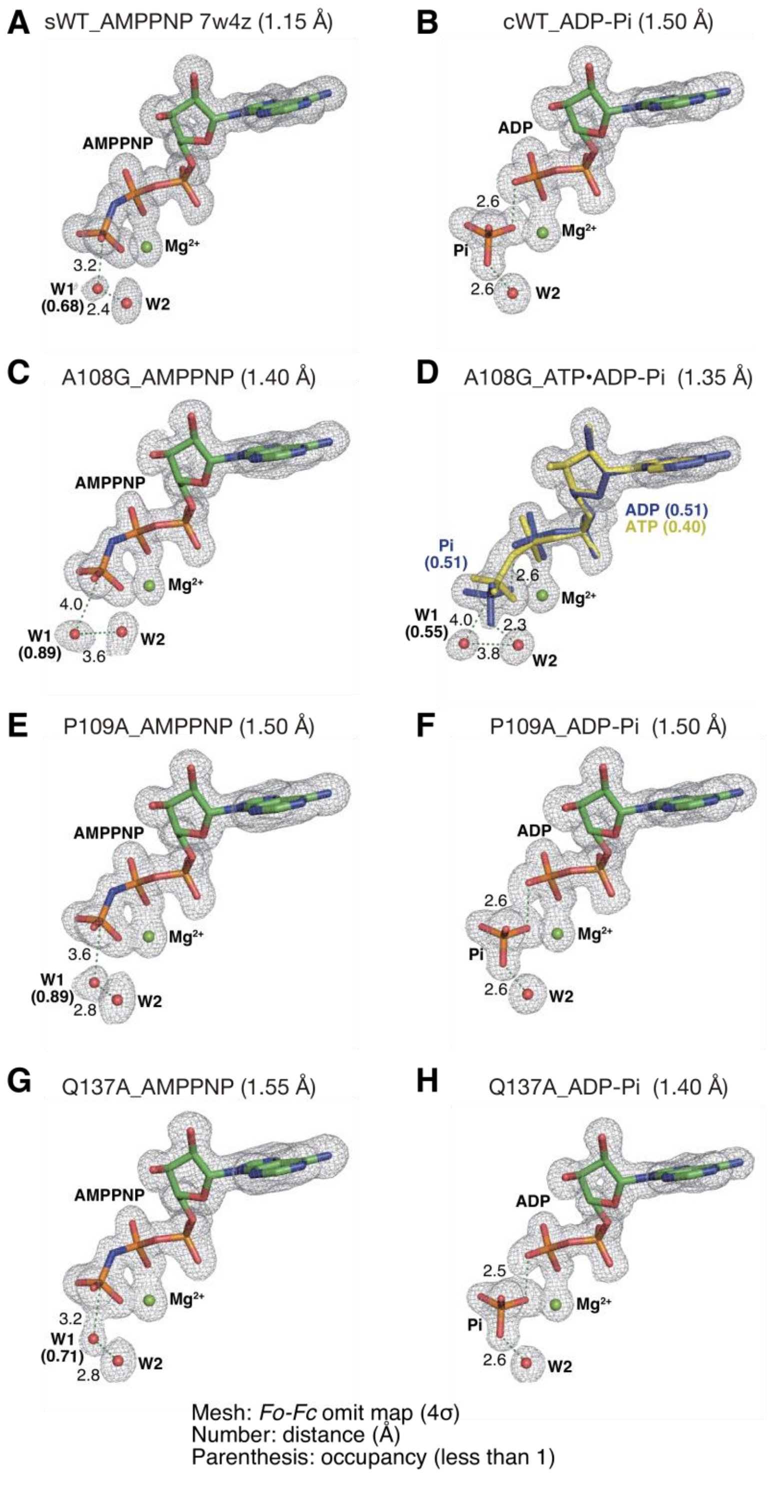

Supplementary Figure S3. Nucleotide structure

Nucleotides and the bound Mg^{2+}, W1, and W2 of wild type and mutant actins are shown with the omit map contoured at 4σ. Distances between nucleotide and water molecules are indicated in Å. Occupancies are shown in parentheses if less than 1. **(A)** sWT_AMPPNP (PDB 7W4Z). **(B)** cWT_ADP-Pi. **(C)** A108G_AMPPNP. **(D)** A108G_ATP•ADP-Pi. **(E)** P109A_AMPPNP. **(F)** P109A_ADP-Pi. **(G)** Q137A_AMPPNP. **(H)** Q137A_ADP-Pi.

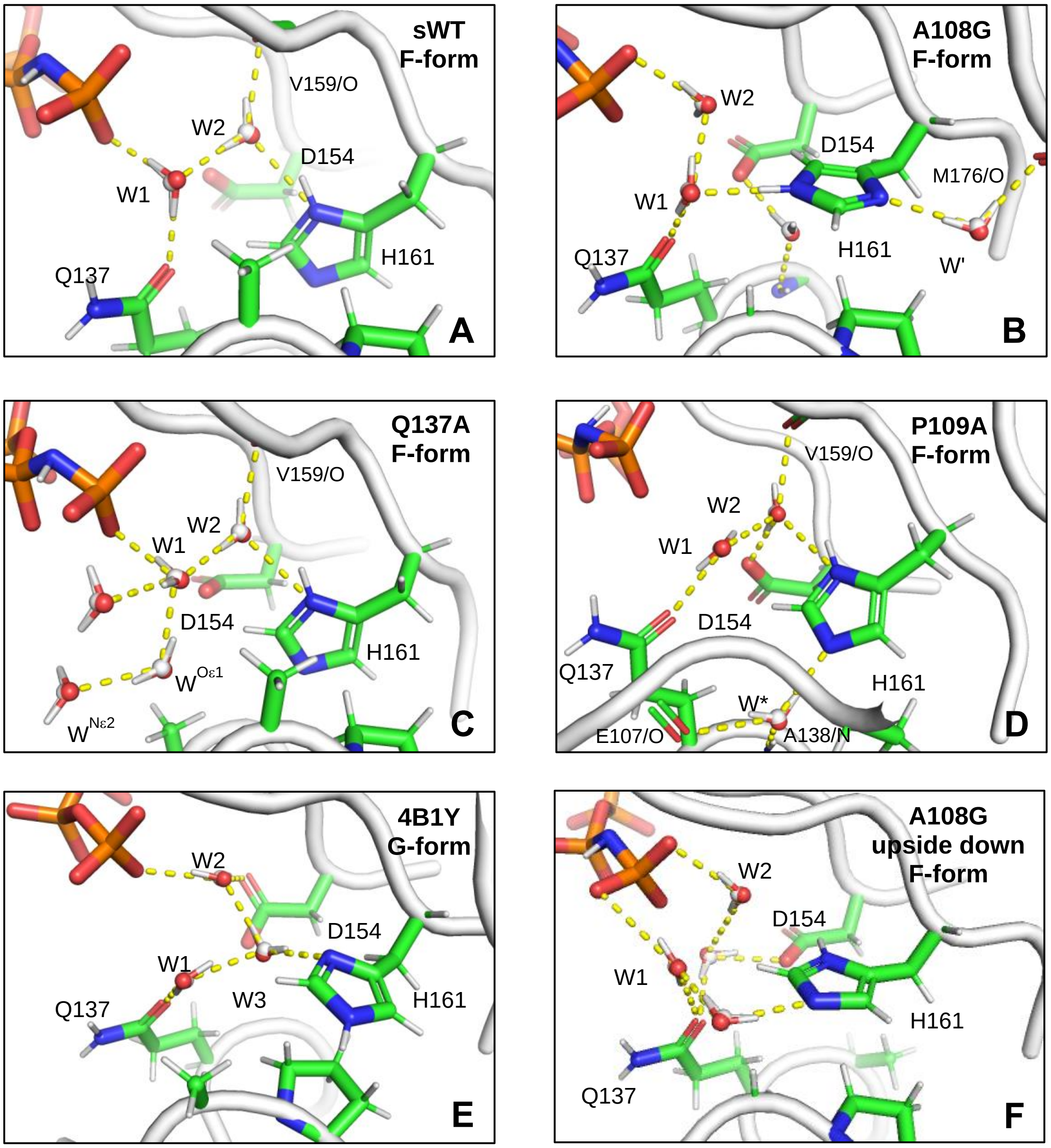

Supplementary Figure S4. Hydrogen bonds network around His 161 in actin.

(A) sWT_AMPPNP with $N^{\delta1}$-protonated H161 (F-form, PDB code: 7W4Z), (B) A108G_AMPNP with $N^{\varepsilon2}$-protonated H161, (C) Q137A_AMPPNP with $N^{\delta1}$-protonated H161, (D) P109A_AMPPNP with $N^{\delta1}$-protonated H161, (E) sWT _ATP (G-form, PDB code: 4B1Y), (F) A108G_AMPPNP with upside down, $N^{\delta1}$-protonated H161. Structures in the figures were made by adding hydrogen atoms and by minimizing the system conformation energy with position restraints for heavy atoms in protein, Mg^{2+} and ligand using the steepest descent method in GROMACS. In other words, only the position and orientation of water molecules in the crystal were optimized. Because of some hydrogen bonds that are too short between water molecules in the crystal from the viewpoint of the force field, the positions of some water molecules shifted, resulting in some local portions of the hydrogen network that do not always match the description of the crystal structure.

A

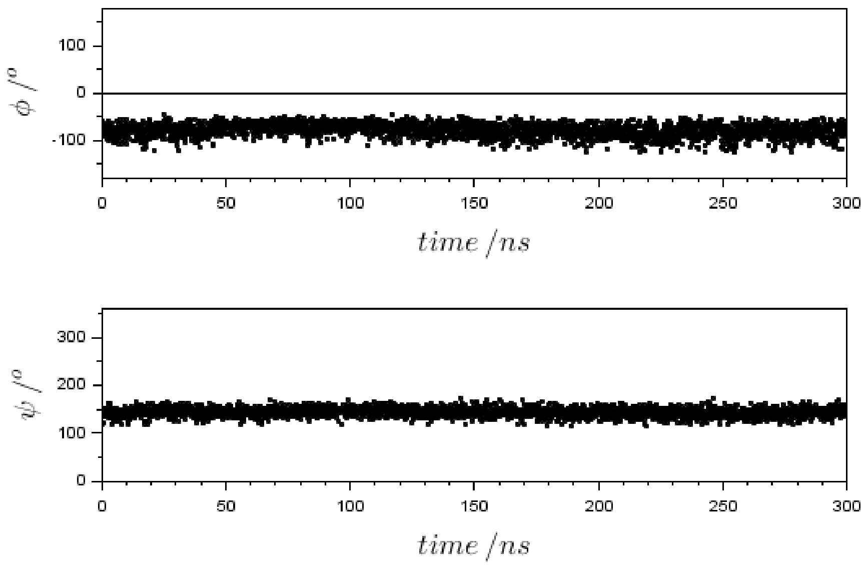

B

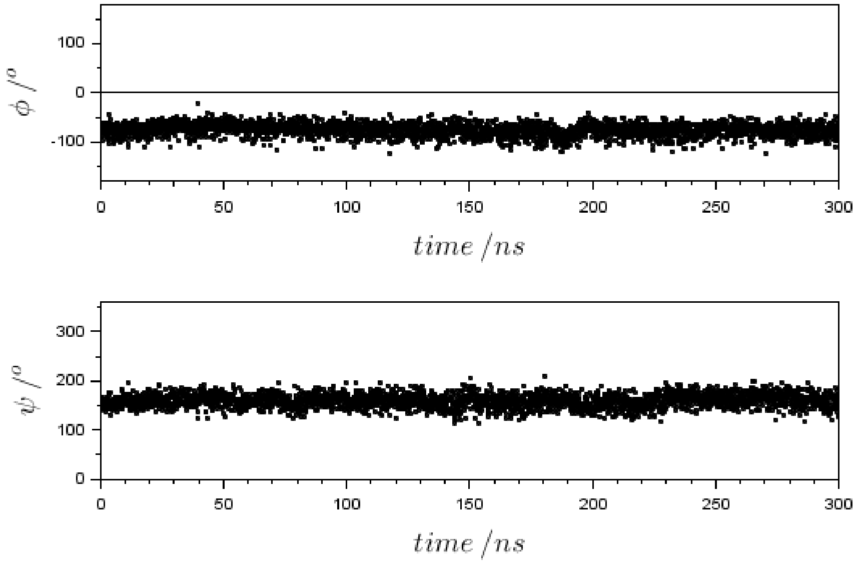

Supplementary Figure S5. Fluctuation of Pro109 due to the mutation of Ala108 to glycine.

Typical time course of the dihedral angles of main chain at 108, ϕ and φ: $\phi = -78 \pm 14^{o}$ (mean $\pm$ SD) and $\varphi = 144 \pm 9^{o}$ in WT **(A)** and $\phi = -76 \pm 13^{o}$ and $\varphi = 159 \pm 14^{o}$ in A108G **(B)**. The angles are independent on conformation of His161. The resulting fluctuation of Pro109 is larger than that in WT. $\phi = -71^{o}$ and $\varphi = 148^{o}$ in the crystal structure of sWT and ϕ = -75º and $\varphi = 153^{o}$ in the crystal structure of A108G.

2 Supplementary Videos

Supplementary Video S1. Water dynamics in the active site in A108G with upside down, $N^{\delta1}$-protonated His161 (δ-tautomer)

The video was made from the MD simulation of A108G with $N^{\delta1}$-protonated H161with position restraints for heavy atoms in protein, Mg^{2+} and AMPPNP for 5 ns. Orange balls show the positions of oxygen atoms of crystal water. His161 cannot form stable hydrogen bonds with surrounding waters.

Supplementary Video S2. Water dynamics in the active site in A108G with $N^{\varepsilon2}$-protonated H161 (ε-tautomer)

The video was made from the MD simulation of A108G with $N^{\varepsilon2}$-protonated H161 with position restraints for heavy atoms in protein, Mg^{2+} and AMPPNP for 5 ns. Orange balls show the positions of oxygen atoms of crystal water. The positions of W2 and W* were maintained.

Supplementary Video S3. Water dynamics in the active site of G-form actin

The video shows water dynamics in the active site of G-form actin (PDB code 4B1Y), including ATP, Ala108, Pro109, Gln137, Asp154, His161, ATP, W1 (red), W2 (orange), and W3 (wheat). The video was made from the MD simulation on G-actin with $N^{\varepsilon2}$-protonated H161 with position restraints for heavy atoms in protein, Mg^{2+} and ATP for 15 ns. In the case of G-actin with $N^{\delta1}$-protonated H161, also, the similar water dynamics were observed, but the shifts of crystal waters were slightly larger than in the case of $N^{\varepsilon2}$-protonated H161. Protonation of H161 cannot be determined without ambiguity.

Supplementary Video S4. Water dynamics in the active site of sWT

The video shows water dynamics the active site of F-form sWT, including ATP, Ala108, Pro109, Gln137, Asp154, His161, ATP, W1 (red), and W2 (orange). The video was made from the MD simulation of sWT with $N^{\delta1}$-protonated H161 with position restraints for heavy atoms in protein, Mg^{2+} and ATP for 15 ns. The protonation of His161 was shown in the previous study (1). The starting structure was built by replacement of N^{3B} (ANPPNP) in sWT_AMPPNP by O^{3B} (ATP).

Supplementary Video S5. Water dynamics in the active site of Q137A

The video shows water dynamics the active site of F-form Q137A, including ATP, Ala108, Pro109, Asp154, His161, ATP, W1 (red), W2 (orange) $W_{O\varepsilon1}$ (blue), and $W_{N\varepsilon1}$ (yellow). The video was made from the MD simulation of Q137A with $N^{\delta1}$-protonated H161 with position restraints for heavy atoms in protein, Mg^{2+} and ATP for 15 ns. The starting structure was built by replacement of N^{3B} (ANPPNP) in Q137_A_AMPPNP by O^{3B} (ATP).

3 Supplementary References

1. Kanematsu Y, Narita A, Oda T, Koike R, Ota M, Takano Y, et al. Structures and mechanism of actin ATP hydrolysis. Proc Natl Acad Sci U S A. 2022;119(43):e2122641119.

2. Iwasa M, Maeda K, Narita A, Maéda Y, Oda T. Dual roles of Gln137 of actin revealed by recombinant human cardiac muscle alpha-actin mutant. J Biol Chem. 2008; 283(30):21045-53

3. Iwasa M, Aihara T, Maeda K, Narita A, Maeda Y, Oda T. Role of the Ala-108-Pro-112 loop in actin polymerization and ATPase activities. J Biol Chem. 2012; 287(30):43270-6

4. Choe H, Burtnick LD, Mejillano M, Yin HL, Robinson RC, Choe S. The calcium activation of gelsolin: insights from the 3A structure of G4-G6/actin complex. J Mol Biol. 2002; 324(4):691-702
